# Magnetic lignin-supported sulfonic acid ionic liquid: a novel catalyst for synthesis of 2-amino-4-aryl-4*H*-benzo[*f*]chromen-3-carbonitrile derivatives

**DOI:** 10.1039/d5ra07067h

**Published:** 2025-11-25

**Authors:** Majidreza Gerami, Mahnaz Farahi, Bahador Karami

**Affiliations:** a Department of Chemistry, Yasouj University Yasouj 75918-74831 Iran farahimb@yu.ac.ir +98 7412242167

## Abstract

A novel Brønsted acidic ionic liquid immobilized on modified magnetic lignin (Fe_3_O_4_/MPL–[IL]) was successfully synthesized and comprehensively characterized by FT-IR, XRD, EDX, VSM, FE-SEM, TGA, and TEM analyses. The prepared nanocatalyst was utilized for preparing 2-amino-4-aryl-4*H*-benzo[*f*]chromene-3-carbonitriles *via* the reaction between aryl aldehydes, β-naphthol, and active methylene compounds (malononitrile or ethyl cyanoacetate), affording high yields of 87–97% within short reaction times (20–80 min) under mild conditions. Necessary experiments were conducted for the recyclability test of the prepared catalyst, and the results showed that the Fe_3_O_4_/MPL–[IL] nanocatalyst can be reused five times while maintaining about 95% of its initial catalytic activity, with excellent stability.

## Abstract 2

A novel Brønsted acidic ionic liquid immobilized on modified magnetic lignin (Fe_3_O_4_/MPL–[IL]) was successfully synthesized and comprehensively characterized by FT-IR, XRD, EDX, VSM, FE-SEM, TGA, and TEM analyses.

## Graphical abstract

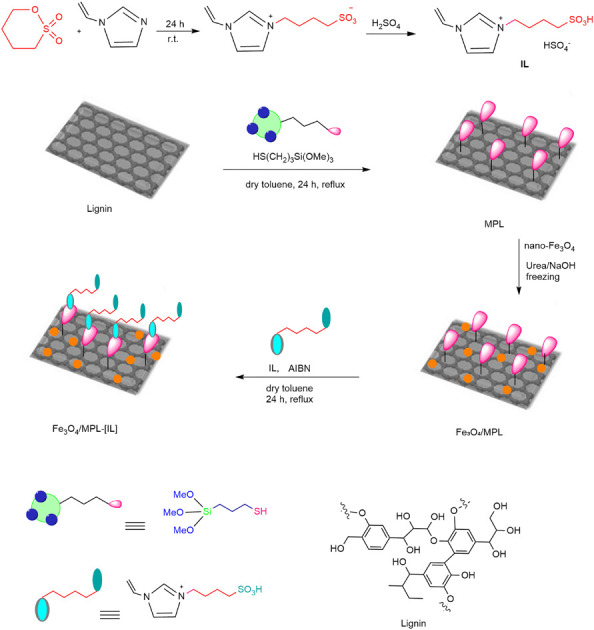

## 1. Introduction

Catalysts play a fundamental role in modern chemical industries by accelerating reactions, improving selectivity, and reducing energy consumption without being consumed in the process.^1–3^ They are indispensable in a wide range of transformations, from fine chemical synthesis to large-scale petrochemical processes.^4–6^ Generally, chemical reactions in homogeneous catalysis include well-known processes such as polymerization,^7–10^ and Hantzsch^11^ reactions using organometallic complexes. However, homogeneous catalysts often require extensive purification after the reaction and cannot be easily recycled, which increases operational costs and environmental impact. In contrast, the use of reusable heterogeneous catalysts could be cost-effective and more eco-friendly, because they can be easily separated from the reaction mixture, regenerated, and reused multiple times without significant loss of activity. This minimizes the need for large amounts of catalyst materials and reduces waste generation, thereby lowering overall production costs and environmental impact. Heterogeneous catalysts play a crucial role in modern chemistry by facilitating selective and efficient chemical transformations. Unlike homogeneous catalysts, they rely on the interaction between solid-phase catalysts and reactants in a separate phase, offering advantages in catalyst recovery, recyclability, and process scalability.^12,13^ To enhance their stability and environmental compatibility, recent advances focus on immobilizing active catalytic species onto natural polymeric substrates such as lignin and cellulose.^14^ Lignin, the second most abundant natural polymer after cellulose, is a complex and heterogeneous aromatic polymer derived from lignocellulosic biomass.^15^ Its high carbon content, thermal stability, and redox-active sites make lignin an ideal candidate for use as a green catalyst or as a support in heterogeneous catalysis. Lignin, as a biopolymer, is rich in functional groups such as hydroxyl, carboxyl, and methoxy groups, allowing for the covalent or non-covalent attachment of catalytic species through functionalization strategies.^16,17^ The immobilization of the catalyst on lignin improves catalyst dispersion, the physicochemical stability of catalysts, decreases leaching under reaction conditions, and allows for efficient recovery and reuse. Moreover, the porous morphology and tunable surface chemistry of lignin contribute to improved mass transfer and catalytic activity. Additionally, this natural support can contribute to catalytic performance through synergistic effects or by creating favorable microenvironments.^18,19^ The supported metal nanoparticles or organometallic complexes onto lignin matrices have shown capable results in biomass valorization,^20^ oxidation,^21^ and hydrogenation reactions.^22^ This approach not only reduces reliance on synthetic polymeric supports but also valorizes underutilized agro-industrial residues. Therefore, this approach aligns with principles of green chemistry and sustainable development by applying low-cost, ecological support and reducing dangerous waste.^23^ As such, natural biopolymers represent a useful platform for engineering robots and eco-friendly heterogeneous catalytic systems.^24^ Although lignin has been widely used as support for the synthesis of reusable supported catalysts, one of the main problems with them is the difficulty of separation from the reaction mixture by filtration and centrifugation. The key to overcoming these limitations is the use of magnetic nanoparticles (MNPs), which allow easy and faster separation of the catalyst from the reaction mixture by an external magnetic field. By integrating lignin into a magnetic framework, this catalyst offers both structural integrity and facile recovery, making it a promising candidate for various catalytic applications.^25,26^

Ionic liquids (ILs) are salts composed of large asymmetric organic cations and inorganic or organic anions that remain in the liquid state at or near room temperature.^27^ Ionic liquids have different properties from molecular liquids, making them favorable materials for use in a diversity of fields. During the past decade, they have been central to many areas of chemistry because of their broad applicability and unique properties such as a wide liquid range, low vapor pressure, good conductivity, high thermal stability, and non-flammability. Their physicochemical properties are largely governed by their special structure and the interaction of ions.^28–30^ Ionic liquids decrease environmental impact by minimizing solvent releases and improving reaction efficiency. Owing to negligible vapor pressures and their non-flammable nature, they have been excellent alternatives to traditional organic solvents in chemical reactions. Their structures can be improved by changing the ions, allowing chemists to design them for specific functions. Furthermore, they are widely used in a variety of applications, such as electrochemistry,^31^ organic synthesis catalysis,^32^ extraction,^33^ and separation processes.^34^ They also show a role in processing biomass by way of dissolving tough materials. Researchers are also exploring their use in drug delivery systems and nanotechnology, where their ability to dissolve a wide range of substances and interact selectively with biomolecules offers exciting potential.^35–39^ One vital application of ionic liquids is that they show wide usage as a catalyst in many organic transformations. However, when employed alone as homogeneous catalysts, ILs may face limitations such as difficult recovery and potential leaching. Therefore, immobilizing ionic liquids on solid substrates, such as magnetic lignin, has emerged as a promising strategy to improve their recyclability and stability in numerous catalytic and separation processes.^40,41^ This synergy often leads to enhanced catalytic activity, better selectivity, and greater process efficiency.^42,43^ A combination of fascinating features of ionic liquid with those of the supporting material will develop novel performances when the synergistic effects appear. ILs have been supported on different solid supports such as MCM-41,^44^ chitosan,^45^ SBA-15,^46^ Merrifield resin,^47^ silica gel,^48^ alumina,^49^ molecular sieves,^50^ clays,^51^ carbon nanotubes,^52^*etc.* The obtained materials are known as supported ionic liquids (SILs). Different methods have been used for IL immobilization, such as the sol–gel method,^53^ impregnation,^54^ encapsulation,^55^ and grafting method.^56^

In recent years, there has been a growing interest in the syntheses of benzo[*f*]chromene and its derivatives because most of the compounds with biological activity are derived from these compounds. Benzo[*f*]chromenes play a fundamental role in both organic chemistry and pharmaceutical development due to their diverse chemical properties and biological activities.^57,58^ Their rigid polycyclic framework also renders them valuable scaffolds in medicinal chemistry and material science. Compounds containing the benzo[*f*]chromene skeleton are known to have antitumor properties.^59^ Moreover, several prospective non-sedative anxiolytic agents with the benzo[*f*]chromene structure have been discovered.^60^ These heterocyclic compounds widely occur in nature in the form of alkaloids, vitamins, pigments, and as constituents of plant and animal cells.^61^ Therefore, several methods have been reported for the preparation of benzo[*f*]chromene derivatives.^62,63^

Following our continuous interest in introducing new and safe heterogeneous catalysts,^64–70^ and by considering all the reasons and importance of clean synthetic procedures, this work presents the design and synthesis of Fe_3_O_4_/MPL-supported Brønsted acidic ionic liquid system as a heterogeneous catalyst, which combines the benefits of easy recovery, eco-friendliness, and cost-effectiveness compared to conventional homogeneous catalysts.^71–73^ The structural and chemical features of the catalyst were thoroughly characterized, and its performance was evaluated in the one-pot synthesis of 2-amino-4-aryl-4*H*-benzo[*f*]chromene-3-carbonitrile derivatives. In addition, the catalyst recyclability and operational stability were examined to assess its practical applicability in green synthetic processes.

## 2. Experimental

### 2.1. Materials and instrumentation

All reagents, solvents, and chemicals were purchased from Merck, Sigma-Aldrich, and Fluka and used without further purification. FT-IR spectra were recorded on a Jasco 6300D spectrophotometer (400–4000 cm^−1^). Melting points were determined using a Kruss Electrothermal KSB1N melting point apparatus. XRD, FE-SEM, TEM, EDX, TGA, and VSM analyses were performed using standard instrumentation (see SI for more details).

### 2.2. Catalyst preparation (Fe_3_O_4_/MPL–[IL])

The Fe_3_O_4_/MPL–[IL] catalyst was prepared by functionalizing Fe_3_O_4_ nanoparticles with 3-mercaptopropyl lignin (MPL), followed by immobilization of the acidic ionic liquid [IL] onto the Fe_3_O_4_/MPL composite. The general trend involved dispersing Fe_3_O_4_ in an aqueous alkaline solution, adding MPL, and subsequently attaching the ionic liquid under reflux in toluene with AIBN as an initiator. Detailed synthetic procedures for Fe_3_O_4_, MPL, [IL], and Fe_3_O_4_/MPL–[IL] are provided in the SI.

### 2.3. General procedure for the synthesis of 2-amino-4-aryl-4*H*-benzo[*f*]chromen-3-carbonitriles using Fe_3_O_4_/MPL–[IL]

A mixture of aldehyde (1 mmol), malononitrile (1 mmol), 2-naphthol (1 mmol), and Fe_3_O_4_/MPL–[IL] (0.004 g) was stirred at 95 °C under solvent-free conditions. When the reaction was complete (monitored by TLC), hot ethanol (10 mL) was added, and the magnetic catalyst was separated using a magnet. The pure product was obtained by recrystallization from ethanol. The catalyst was reused directly in subsequent reactions after washing with water and ethanol and drying, with no loss of activity.

### 2.4. Safety and handling precautions

All ionic liquids and nanocatalysts were handled following standard laboratory safety protocols. The ionic liquid was determined to be non-volatile and non-flammable; however, gloves were used, and work was conducted in a well-ventilated fume hood to avoid direct contact with skin or inhalation of vapors. The magnetic nanocatalyst was handled carefully to prevent inhalation of fine particles and accidental ingestion. No specific hazards were observed during the synthesis or catalytic reactions.

## 3. Results and discussion

**Scheme 1:**
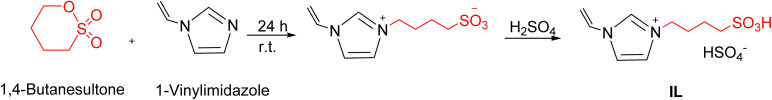
Preparation of the ionic liquid (IL).

**Scheme 2:**
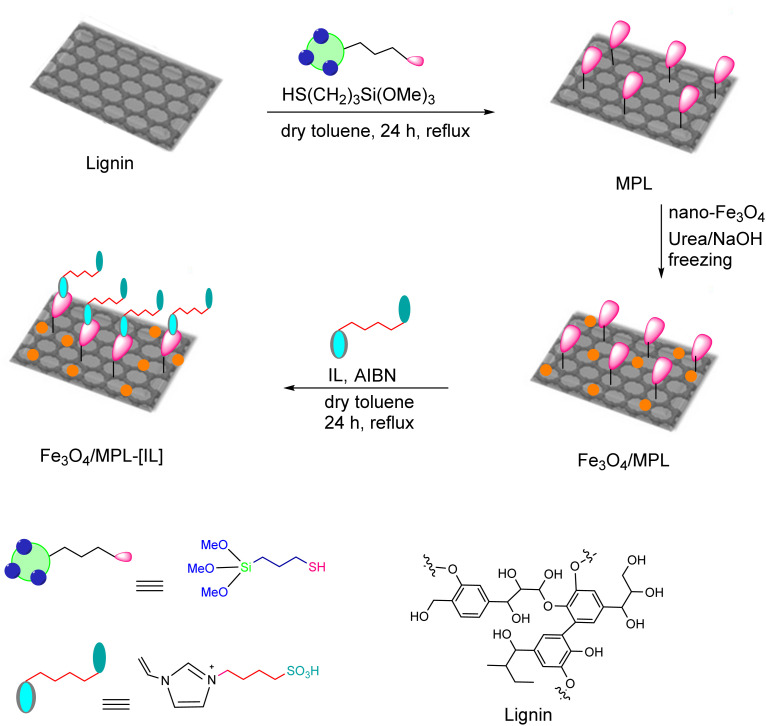
Preparation of Fe_3_O_4_/MPL–[IL] nanocatalyst.

This study presents the synthesis and characterization of Fe_3_O_4_/MPL–[IL], a catalyst with notable efficiency, and its use in producing 2-amino-4-aryl-4*H*-benzo[*f*]chromen-3-carbonitrile derivatives. The step-by-step procedure for creating magnetic nanoparticles functionalized with an acidic ionic liquid is illustrated in Schemes 1 and 2. The process began with the modification of lignin using 3-mercaptopropyltrimethoxysilane, forming siloxane-based bonds to yield 3-mercaptopropyl lignin (MPL). Subsequently, Fe_3_O_4_/MPL was obtained by chemical modification of Fe_3_O_4_ nanoparticles with MPL in an alkaline urea/NaOH environment. The ionic liquid [IL] was synthesized by reacting 1-vinylimidazole with 1,4-butanesultone, followed by treatment with sulfuric acid (Scheme 1).

Finally, the Fe_3_O_4_/MPL–[IL] was produced *via* radical copolymerization between IL and Fe_3_O_4_/MPL (Scheme 2). The resulting nanocomposite was analyzed by various characterization techniques, including FT-IR, XRD, EDX, VSM, FE-SEM, TGA, and TEM analysis.

**Fig. 1:**
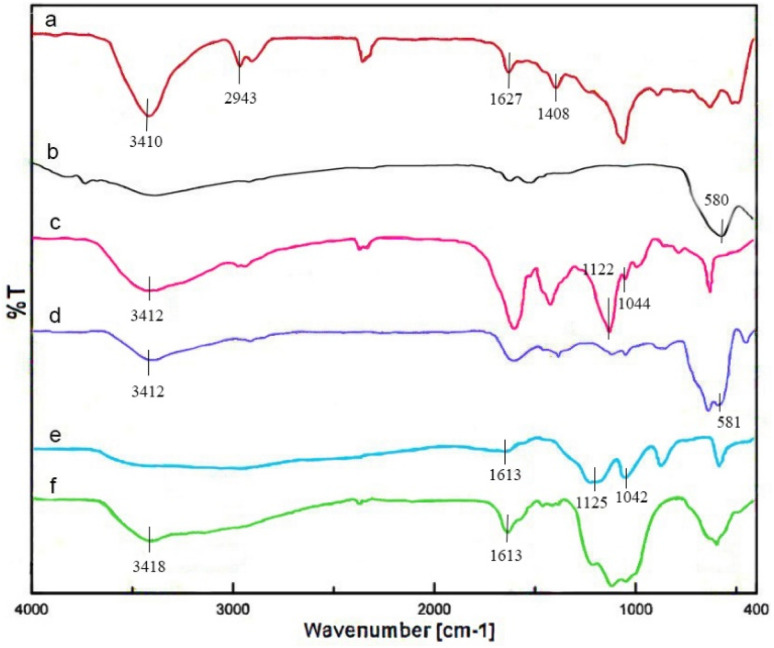
The FT-IR spectrum of (a) lignin, (b) Fe_3_O_4_, (c) MPL, (d) Fe_3_O_4_/MPL, (e) [IL], and (f) Fe_3_O_4_/MPL–[IL].

The synthesis of Fe_3_O_4_/MPL–[IL] nanocatalyst was monitored using FT-IR spectroscopy (Fig. 1). As illustrated in Fig. 1a, broad absorption near 3410 cm^−1^ signifies the O–H stretching of phenolic groups inherent to the lignin matrix. The signal at 2943 cm^−1^ corresponds to vibrations of aliphatic C–H bonds. A band at 1627 cm^−1^ evidences the presence of carbonyl groups. Aromatic C=C stretching vibrations appear at 1408 cm^−1^, confirming the benzene ring structure (Fig. 1a).^74^ The FT-IR spectrum of magnetite (Fe_3_O_4_) is characterized by absorption bands in the low-wavenumber region, which arise from lattice vibrations of metal–oxygen bonds. A strong and well-defined band typically appears at approximately 570–590 cm^−1^ corresponding to the stretching vibrations of Fe–O bonds (Fig. 1b).^75^ In Fig. 1c, characteristic bands for symmetric and asymmetric Si–O stretching are found at 1044 and 1122 cm^−1^, respectively, and a peak indicative of S–H bonds is observed at 2550 cm^−1^.^76^ The presence of the Fe–O peak in all samples implies that Fe_3_O_4_ nanoparticles were retained throughout modification steps (Fig. 1d and f). In Fig. 1e, the absorption bands at 1613 cm^−1^ correspond to the stretching vibration of a C=N band, and the peaks around 3154 cm^−1^ is related to sp^2^ C–H of imidazolium ring. Vibrational bands located at 1042 cm^−1^ and 1125 cm^−1^ are attributed to C–S and S=O bonds, which confirms the existence of the –SO_3_H group (Fig. 1e).^77^ Notably, in the FT-IR spectrum of the final Fe_3_O_4_/MPL–[IL] nanocatalyst (Fig. 1f), several diagnostic absorption bands confirm the successful integration of the composite constituents. The signals located at approximately 2945 cm^−1^ and 3418 cm^−1^ are attributed to the stretching vibrations of aliphatic –CH_2_ groups and hydroxyl functionalities, respectively, which indicate the presence of lignin-derived structural units as well as surface –OH groups originating from the modification steps. A distinct absorption at 1613 cm^−1^ corresponds to the C=N stretching vibration, providing clear evidence for the incorporation of the imidazolium-based ionic liquid into the hybrid structure. The simultaneous presence of Fe–O vibrational modes alongside these organic functional groups confirms that Fe_3_O_4_ nanoparticles remained intact throughout the modification process and were effectively stabilized within the lignin/ionic liquid matrix. Collectively, these spectral features demonstrate the preservation of lignin's inherent functionalities, the successful anchoring of ionic liquid moieties, and the structural integrity of Fe_3_O_4_, thereby validating the synthesis of the targeted Fe_3_O_4_/MPL–[IL] nanocatalyst.

**Fig. 2:**
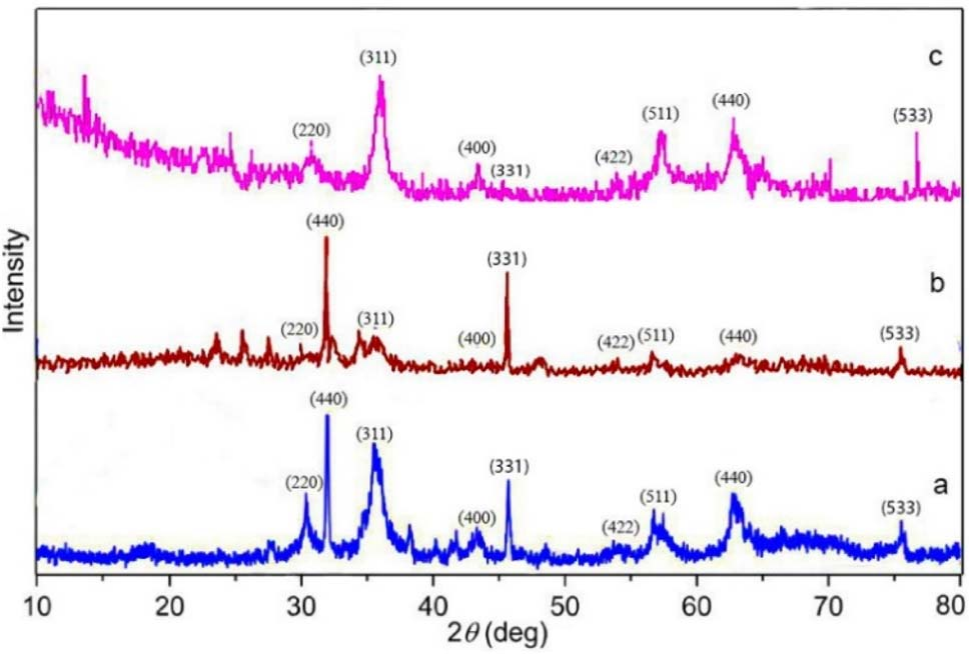
XRD patterns of (a) Fe_3_O_4_, (b) Fe_3_O_4_–lignin, and (c) Fe_3_O_4_/MPL–[IL].

XRD analysis was used to examine the structural properties of Fe_3_O_4_, Fe_3_O_4_–lignin, and the Fe_3_O_4_/MPL–[IL] (Fig. 2). For Fe_3_O_4_ (Fig. 2a), six prominent peaks appeared at 2*θ* values of 30.27°, 35.6°, 42.9°, 53.61°, 57.12°, and 63.31°, corresponding to the (220), (311), (400), (422), (511), and (440) planes, respectively, confirming the formation of a pure spinel crystal structure.^78^ The XRD pattern of the Fe_3_O_4_–lignin composite structure, as shown in Fig. 2b, closely matches previous research findings on Fe_3_O_4_-coated lignin structures. This pattern displays six distinct peaks at 30.28°, 35.6°, 43.3°, 53.57°, 57.47°, and 63.31°, which correspond respectively to the (220), (311), (400), (422), (511), and (440) crystallographic planes.^79^ The XRD pattern for the Fe_3_O_4_/MPL–[IL] catalyst (Fig. 2c) demonstrates significant changes upon functionalization. The structural fingerprint of lignin was altered after modification with layered materials and incorporation of Fe_3_O_4_. Comparison with the earlier spectra (Fig. 2a and b) verifies that Fe_3_O_4_ nanoparticles were successfully integrated and stabilized with ionic liquid (IL) on the lignin matrix, confirming the successful synthesis of the desired nanocatalyst.

**Fig. 3:**
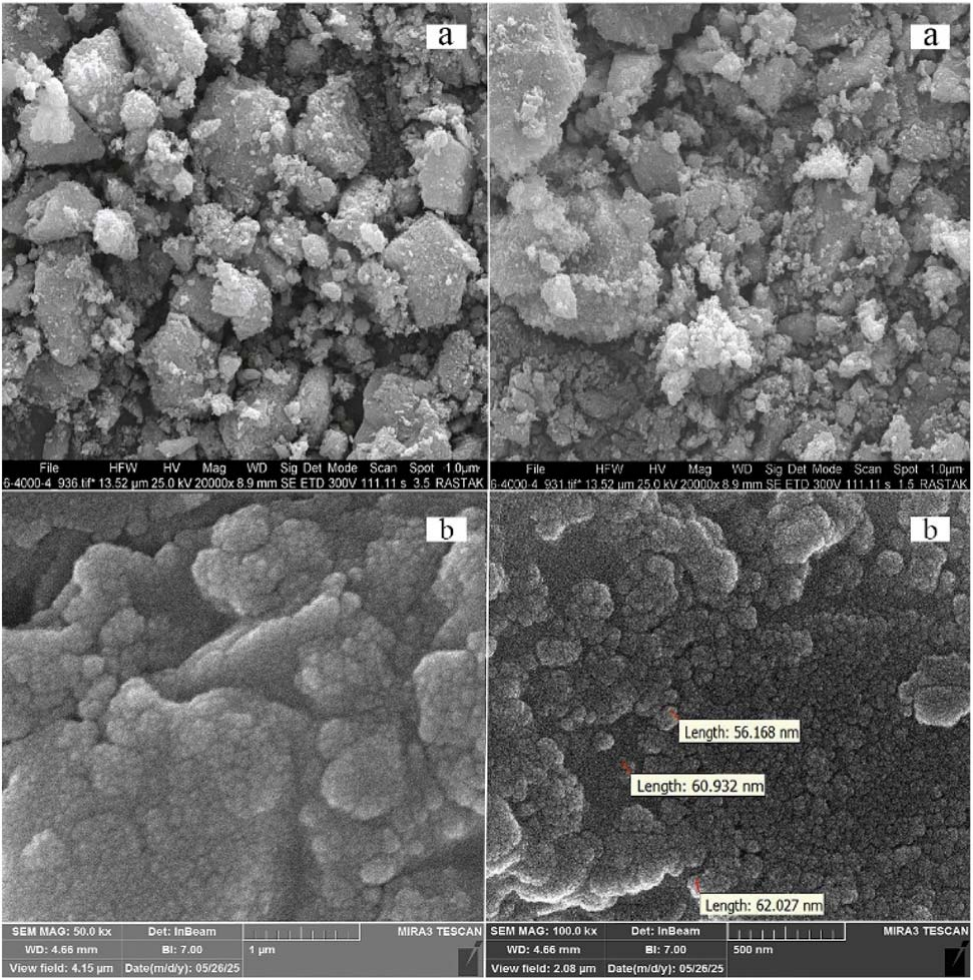
The FE-SEM images of Fe_3_O_4_ (a) Fe_3_O_4_/MPL–[IL] nanocatalyst (b).

The surface morphology and particle size of the Fe_3_O_4_/MPL–[IL] nanocatalyst were evaluated using FE-SEM images (Fig. 3). According to the FE-SEM images, the prepared Fe_3_O_4_/MPL–[IL] nanocatalyst has a spherical morphology and uniform particle distribution, with an orderly size of less than 70 nm. The SEM image of pure Fe_3_O_4_ nanoparticles exhibits a nearly spherical morphology with uniform size distribution. This confirms that the Fe_3_O_4_ core structure remains stable during the subsequent MPL and IL modification processes used in the Fe_3_O_4_–MPL/IL catalyst synthesis.

**Fig. 4:**
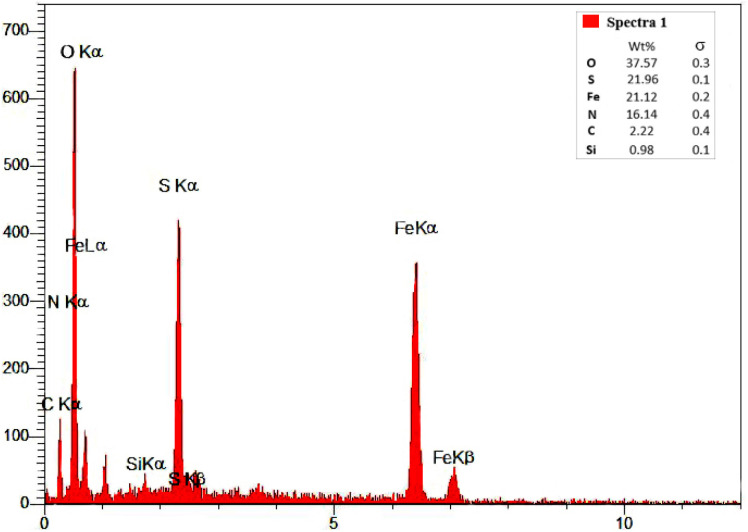
The EDX spectra of Fe_3_O_4_/MPL–[IL] nanocatalyst.

To gain insight into the chemical composition of the Fe_3_O_4_/MPL–[IL] nanoparticles, an EDX analysis was performed, and the results are shown in Fig. 4. The spectrum reveals a prominent signal for iron (Fe), identifying it as the primary metallic constituent. A notable oxygen (O) peak also appears, reflecting a high presence of oxygen-containing groups. Additionally, the signals corresponding to carbon (C), sulfur (S), silicon (Si), and nitrogen (N) are detected. These non-metallic elements likely originate from the lignin component, supporting the effective attachment of both lignin and ionic liquid onto the ferrite nanoparticles.

**Fig. 5:**
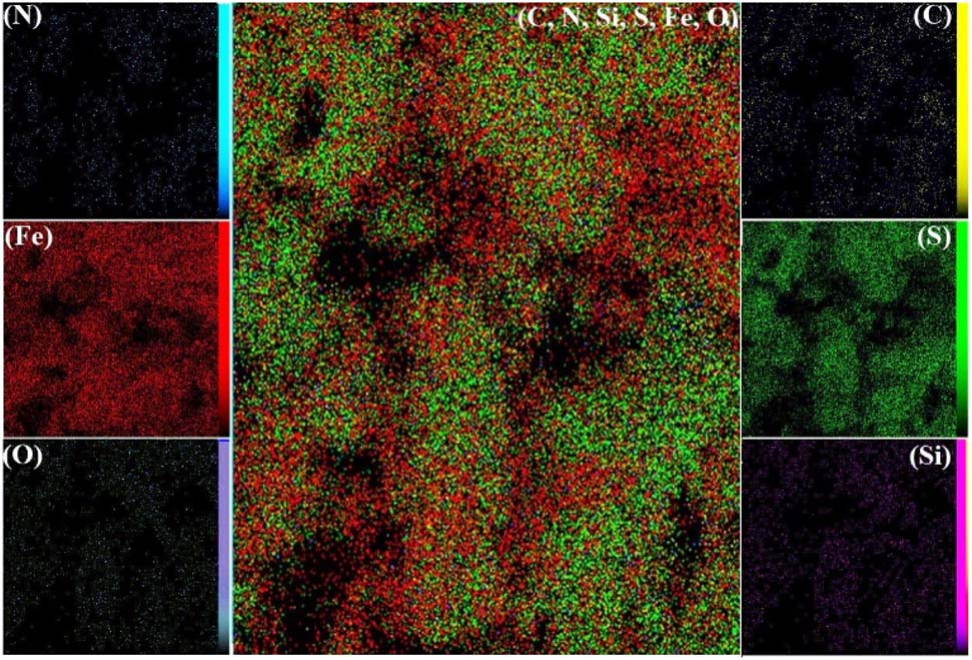
Elemental mapping analysis of Fe_3_O_4_/MPL–[IL] nanocatalyst.

Fig. 5 presents the elemental mapping results for the Fe_3_O_4_/MPL–[IL] nanocatalyst. The distribution maps clearly demonstrate that all key elements, carbon (C), silicon (Si), iron (Fe), oxygen (O), sulfur (S), and nitrogen (N), are evenly dispersed across the catalyst's surface. This uniform elemental spread strongly supports the effective incorporation and stable anchoring of the intended components onto the lignin matrix during the synthesis process.

**Fig. 6:**
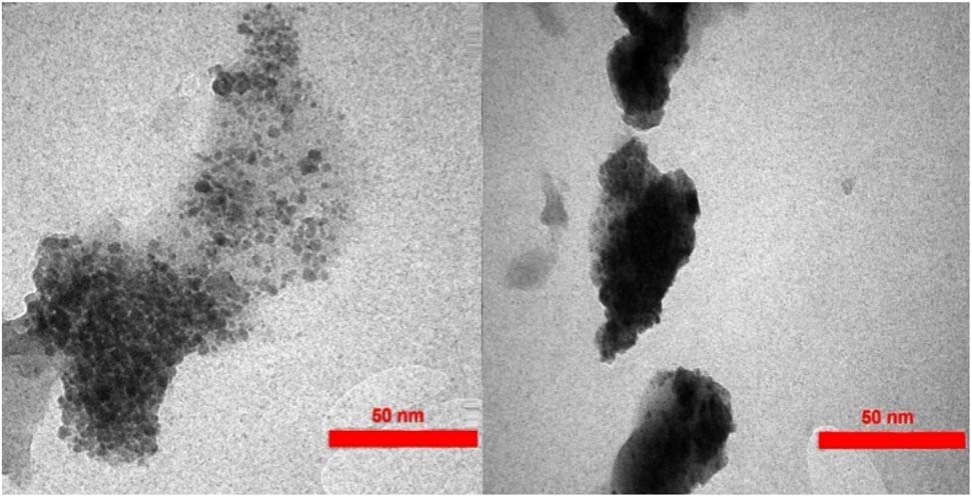
TEM images of Fe_3_O_4_/MPL–[IL] nanocatalyst.

Fig. 6 shows TEM images of the Fe_3_O_4_/MPL–[IL] nanocatalyst. These images reveal magnetite NPs with black cores surrounded by a gray shell of modified lignin. Additionally, the images show that the nanoparticles mainly consist of small, nearly spherical particles.

**Fig. 7:**
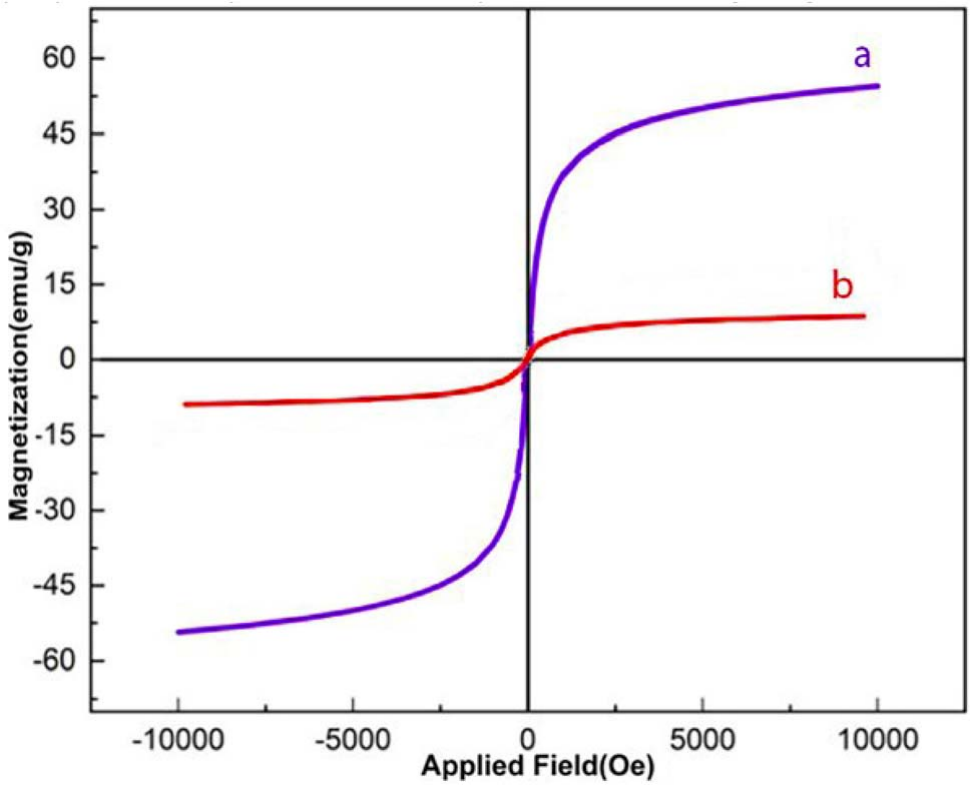
VSM curves of the (a) Fe_3_O_4_ and (b) Fe_3_O_4_/MPL–[IL] nanocatalyst.

The magnetic behavior of the prepared nanocatalyst was examined by vibrating sample magnetometry (VSM), as depicted in Fig. 7. According to the VSM curves, the magnetic saturation values for Fe_3_O_4_ and Fe_3_O_4_/MPL–[IL] composite are 52 and 8 emu g^−1^, respectively. This reduction in magnetization is attributed to the presence of a lignin coating and the incorporation of an ionic liquid, which contributes to the overall decrease in magnetic intensity compared to the uncoated magnetic nanoparticles. However, the magnetic sensitivity of the nanocatalyst is sufficient for its easy magnetic recovery from various reaction mixtures. Therefore, the prepared catalyst can be readily recovered using magnets.

**Fig. 8:**
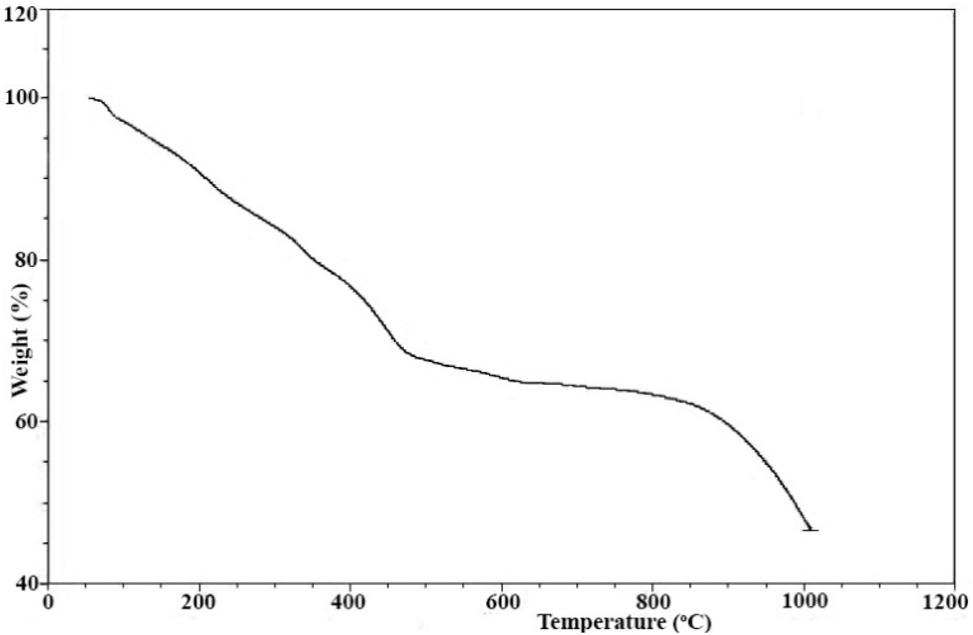
TGA analysis of Fe_3_O_4_/MPL–[IL] nanocatalyst.

The thermal behavior of the Fe_3_O_4_/MPL–[IL] nanocatalyst was assessed through thermogravimetric analysis (TGA), as shown in Fig. 8. The measurements were conducted over a temperature range of 20 to 1000 °C. The resulting TGA profile reflects the structural stability of the material and confirms the presence of functional groups anchored to the nanostructure. An initial mass loss of approximately 4% occurs below 150 °C, which is attributed to the release of physically adsorbed moisture, surface-bound hydroxyl species, and interstitial water molecules. A more pronounced weight reduction, approximately 25%, is observed between 150 °C and 550 °C, likely due to the thermal decomposition of organic moieties, including amine functionalities and sulfate groups attached to the catalyst surface. Finally, a smaller weight decrease of about 6% in the 550–1000 °C range is linked to the breakdown of covalently bonded organic groups on the lignin framework, further indicating the thermal robustness of the synthesized nanocatalyst.

**Scheme 3:**
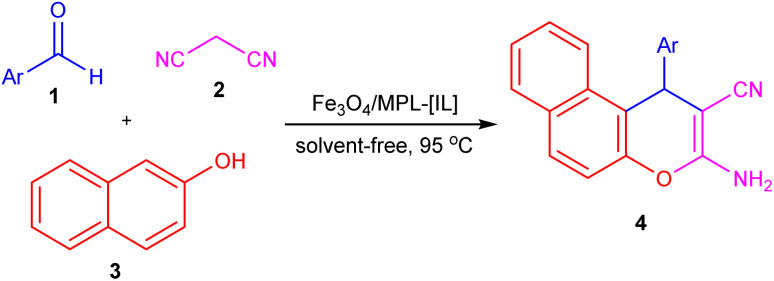
Synthesis of 2-amino-4-aryl-4*H*-benzo[*f*]chromen-3-carbonitriles 4 using Fe_3_O_4_/MPL–[IL] nanocatalyst.

The efficiency of the Fe_3_O_4_/MPL–[IL] nanocatalyst, after its properties were identified, was assessed in producing 2-amino-4-aryl-4*H*-benzo[*f*]chromen-3-carbonitriles 4*via* a reaction between aromatic aldehydes 1, malononitrile 2, and 2-naphthol 3 (Scheme 3).

Initially, to optimize the conditions, the reaction between benzaldehyde (1 mmol), malononitrile (1 mmol), and 2-naphthol (1 mmol) was selected as a model system (Table 1). Without the catalyst, only low yields were achieved, even at higher temperatures, emphasizing the essential role of catalysis in this process. Catalyst loading testing revealed that 0.004 g was optimal, yielding the highest yield at 80 °C. Raising the temperature to 95 °C with this catalyst amount further increased the yield to 97%. Then, the effect of various solvents was investigated to test the model reaction, and the highest yield was observed under solvent-free conditions. Overall, the optimal conditions are 0.004 g of Fe_3_O_4_/MPL–[IL] at 95 °C in a solvent-free environment, yielding excellent catalytic performance. Building on these results, a range of aryl-substituted aldehydes was employed to synthesize a series of 2-amino-4-aryl-4*H*-benzo[*f*]chromen-3-carbonitrile derivatives. According to the data presented in Table 2, these compounds were formed in good to excellent yields.

**Table 1:** Optimization of the reaction conditions for the synthesis of 4a^a^

Entry	Catalyst loading (g)	Solvent	Temp. (°C)	Yield^b^ (%)
1	Cat. 1 (—)	—	25	—
2	Cat. 1 (—)	—	80	10
3	Cat. 1 (0.002)	—	80	60
4	Cat. 1 (0.003)	—	80	75
5	Cat. 1 (0.004)	—	80	80
6	Cat. 1 (0.005)	—	80	70
7	Cat. 1 (0.004)	—	70	80
8	Cat. 1 (0.004)	—	95	97
9	Cat. 1 (0.004)	—	100	90
10	Cat. 1 (0.004)	EtOH	Reflux	80
11	Cat. 1 (0.004)	H_2_O	95	70
12	Cat. 1 (0.004)	Methanol	Reflux	80
13	Cat. 1 (0.004)	Toluene	95	60
14	Cat. 1 (0.004)	EtOH/H_2_O	95	75
15	Fe_3_O_4_ (0.004)	Solvent-free	95	—
16	Fe_3_O_4_/MPL (0.004)	Solvent-free	95	55
17	IL (0.004)	Solvent-free	95	40

a Reaction conditions: benzaldehyde (1 mmol), malononitrile (1 mmol), 2-naphthol (1 mmol). Time: 40 min.

b Isolated yields.

**Table 2:** Synthesis of compound 4 in the presence of Fe_3_O_4_/MPL–[IL] nanocatalyst^a^

Entry	Product 4	M.p. (°C)	Yield^b^ (%)	TON^c^	TOF^d^
4a	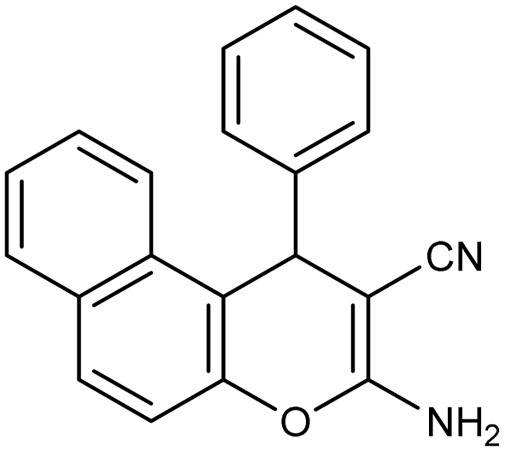	282–284 (ref. 80)	97	122	3.05
4b	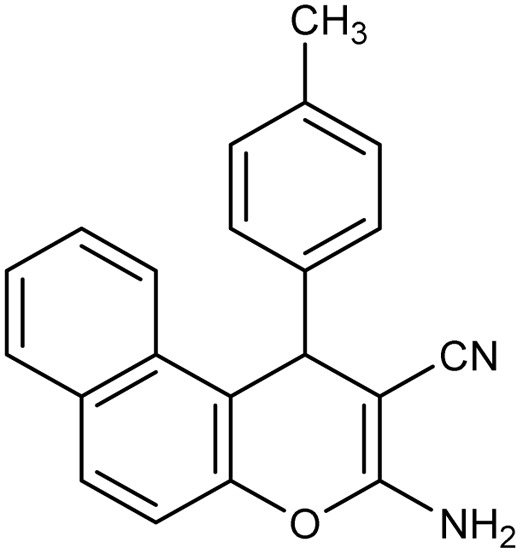	241–243 (ref. 80)	89	112	2.03
4c	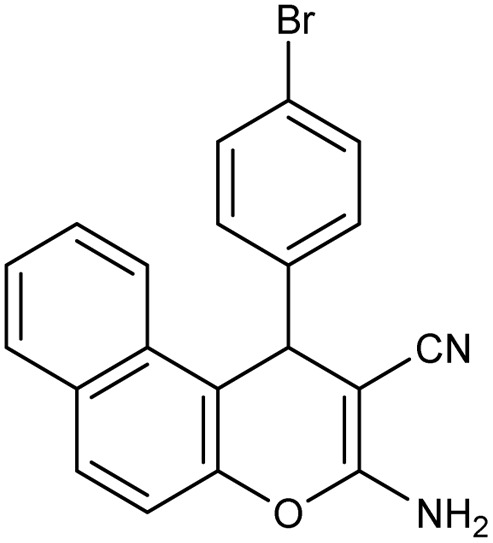	261–263 (ref. 80)	96	121	3.02
4d	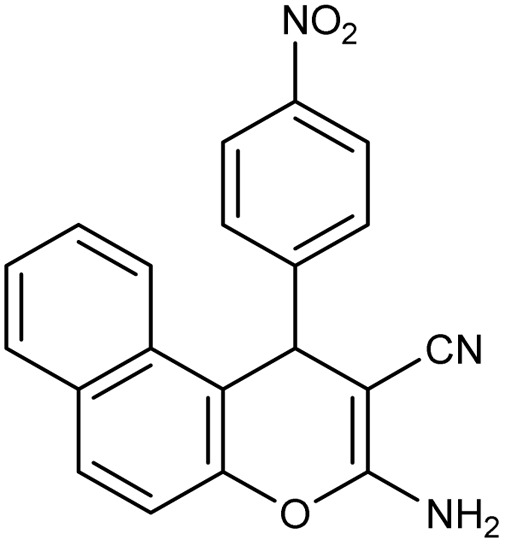	183–185 (ref. 80)	97	122	3.05
4e	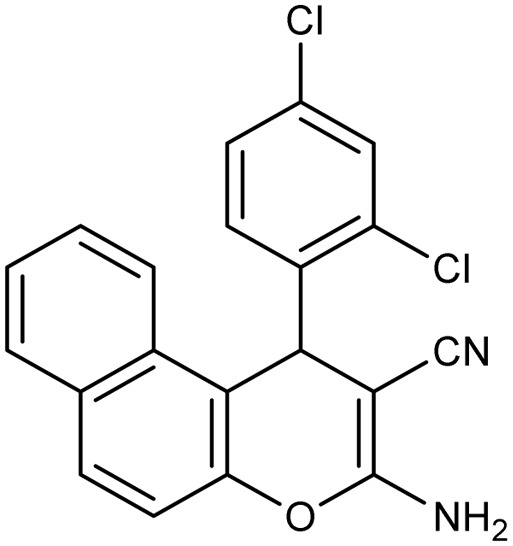	240–242 (ref. 81)	95	119	2.97
4f	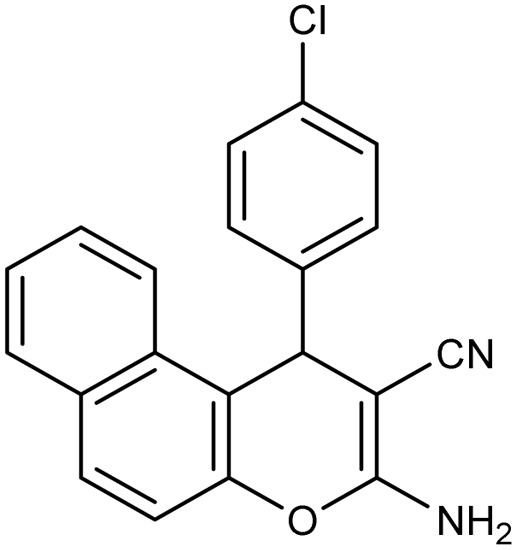	255–257 (ref. 82)	96	121	3.02
4g	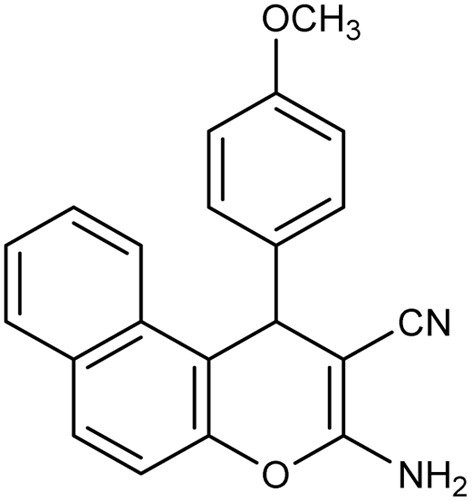	209–211 (ref. 82)	87	109	1.81
4h	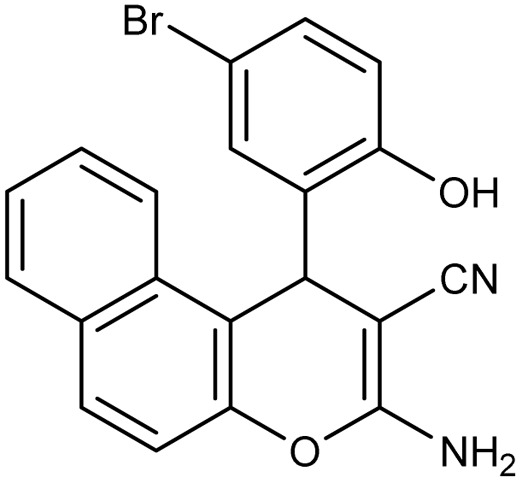	259–261 (ref. 82)	88	110	1.83
4i	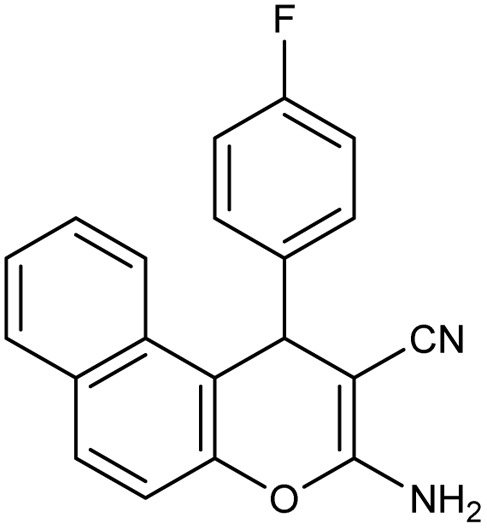	238–240 (ref. 82)	92	116	2.57
4j	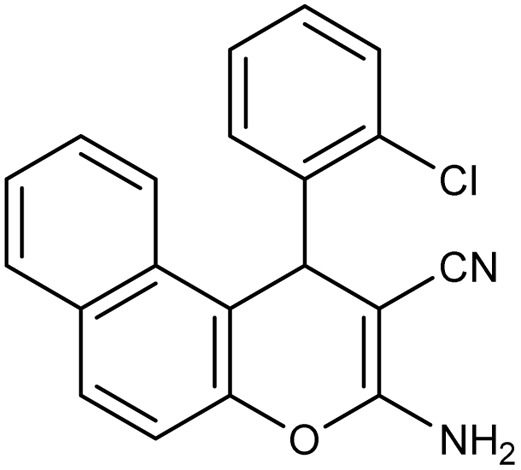	250–252 (ref. 82)	95	119	2.97
4k	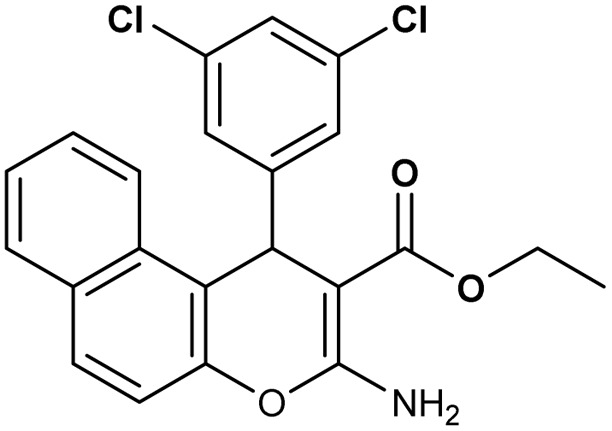	173–175 (ref. 83)	96	121	4.84
4l	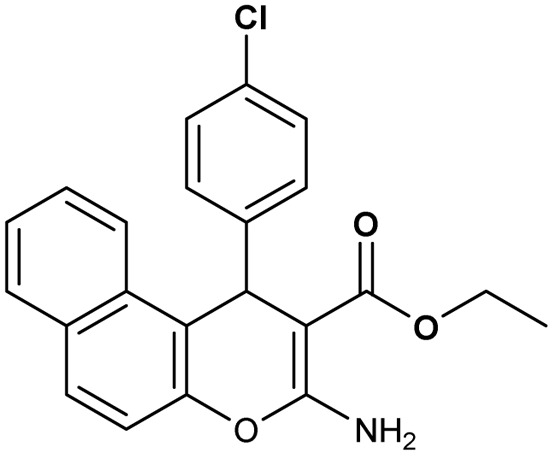	141–143 (ref. 83)	95	119	4.76
4m	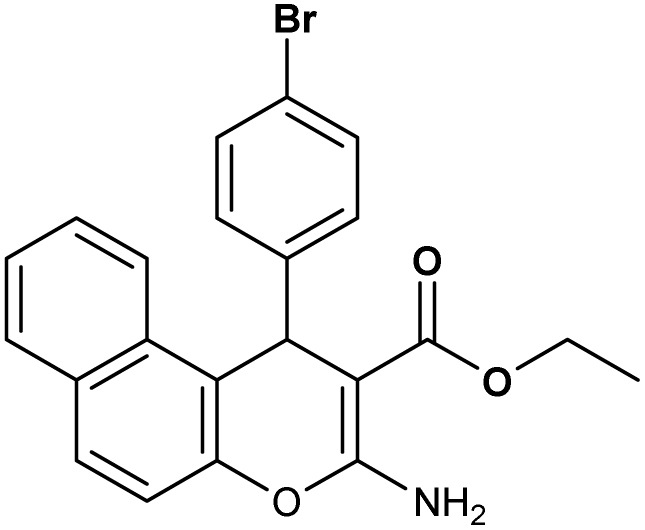	150–152 (ref. 84)	95	119	4.76

a Reaction conditions: aldehyde (1 mmol), malononitrile or ethyl cyanoacetate (1 mmol), 2-naphthol (1 mmol), Fe_3_O_4_/MPL–[IL] (0.004 g), 95 °C, 20–80 min.

b Isolated yields.

c Turnover number [all TONs were calculated by this equation: yield (%)/Cat. (mol%)].

d Turnover frequency [all TOFs were calculated by this equation: TON/time (min)].

**Scheme 4:**
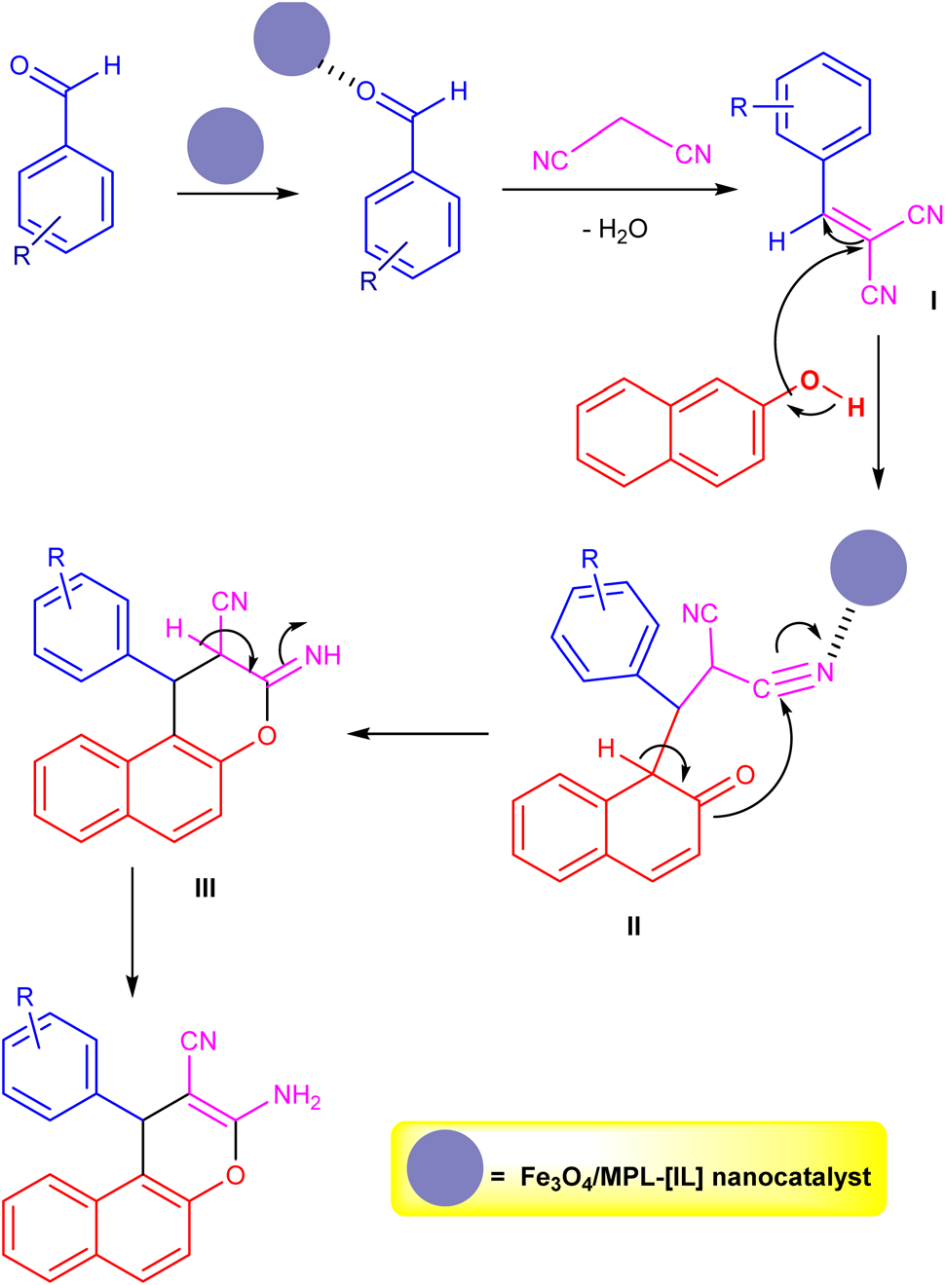
Proposed mechanism for the synthesis of compound 4 using Fe_3_O_4_/MPL–[IL] nanocatalyst.

Based on the findings, Scheme 4 illustrates a proposed pathway for the reaction. Initially, Fe_3_O_4_/MPL–[IL] magnetic nanoparticles serve as an acidic nanocatalyst to activate the aldehyde's carbonyl group, facilitating the formation of α,β-unsaturated intermediate I (2-benzylidene malononitrile) through a Knoevenagel condensation.^85^ Subsequently, a Michael addition between 2-naphthol and intermediate I yields intermediate II. Finally, the intramolecular cyclization of the adduct II to intermediate III and its tautomerization (1,3-H shift) gives the corresponding products.

**Fig. 9:**
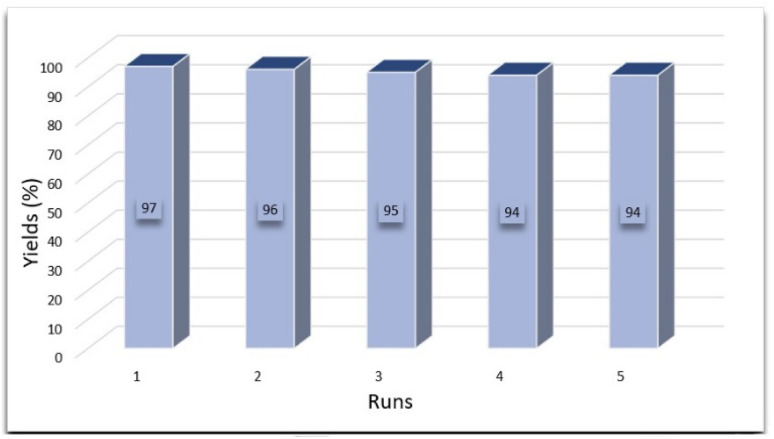
Reusability of Fe_3_O_4_/MPL–[IL] nanocatalyst in the synthesis of 4a.

The potential for reusing Fe_3_O_4_/MPL–[IL], an aspect critical from both environmental and cost perspectives, was investigated in the reaction of benzaldehyde, malononitrile, and 2-naphthol under optimum reaction conditions. Upon completion of the reaction, hot ethanol was added, and the catalyst was isolated from the mixture by employing a magnetic field. It was then rinsed with water and ethanol, dried, and applied again in subsequent reactions. The effectiveness of Fe_3_O_4_/MPL–[IL] over multiple reaction cycles was evaluated (Fig. 9). It maintained substantial catalytic function over five consecutive uses, demonstrating its structural integrity. To examine whether Fe_3_O_4_/MPL–[IL] operates in a homogeneous or heterogeneous manner, a filtration test has been done in the model reaction under optimized reaction conditions. After nearly 50% of the reaction progress, we separated the Fe_3_O_4_/MPL–[IL] catalyst. Then, the mixture residue continued under optimal conditions, but no substantial increase in product conversion was observed. In this regard, no considerable reaction progress was observed, indicating that the active catalytic centers were not washed from the support during the reaction, and the catalyst most likely worked in a heterogeneous manner.

**Fig. 10:**
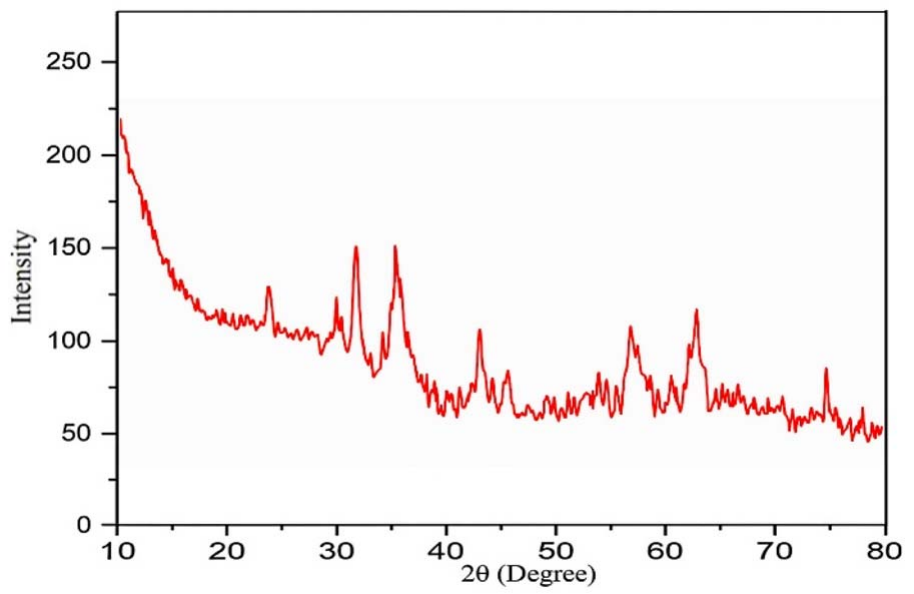
XRD pattern of the recycled Fe_3_O_4_/MPL–[IL] nanocatalyst.

**Fig. 11:**
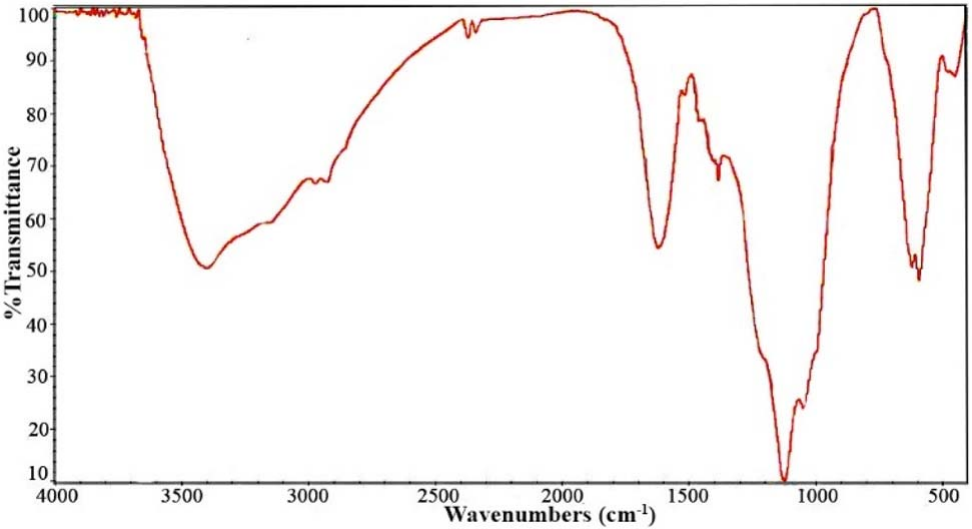
FT-IR spectrum of the recovered Fe_3_O_4_/MPL–[IL] nanocatalyst.

The crystallographic characteristics of the reused catalyst were analyzed through XRD pattern evaluation (Fig. 10). The consistent peak positions and their relative intensities validate the preservation of the catalyst's structure. Furthermore, Fig. 11 displays the FT-IR spectrum of the recycled Fe_3_O_4_/MPL–[IL] nanocatalyst. This spectral data confirms that the structural integrity of the catalyst remains intact even after five cycles of use.

Additionally, the catalytic efficiency of Fe_3_O_4_/MPL–[IL] was compared to previously reported catalysts for the same transformation. As shown in Table 3, this nanocatalyst shows comparable efficiency to other catalysts in terms of reaction times, conditions, and product yield.

**Table 3:** The comparison study between the efficiency of the current catalyst and that of other catalysts in the preparation of 4a

Entry	Catalyst	Conditions	Time (min)/yield^a^ (%)
1	*N*-Octyl phenylphosphinate	Fluorene 0.6 mmol, O_2_ balloon, 130 °C	900/72 (ref. 86)
2	ZSM	H_2_O_2_, H_2_O, 100 °C	240/52 (ref. 87)
3	Au–Pd	H_2_O, MeOH, H_2_O_2_, 120 °C	480/94 (ref. 88)
4	Mg/Al HT	0.1 g, EtOH, r.t.	60/87 (ref. 89)
5	Sodium dodecyl sulfate	0.05 mmol, H_2_O, reflux	60/87 (ref. 90)
6	Fe_3_O_4_/MPL–[IL]	Solvent-free, 95 °C	40/97^b^

a Isolated yields.

b This work.

## 4. Conclusions

In this research, magnetic lignin was utilized as a solid support for anchoring an ionic liquid. The resulting catalyst, denoted as Fe_3_O_4_/MPL–[IL], was thoroughly analyzed using a variety of techniques, including XRD, FE-SEM, TEM, VSM, EDX, TGA, and FT-IR. The catalytic performance of Fe_3_O_4_/MPL–[IL] was evaluated in the preparation of 2-amino-4-aryl-4*H*-benzo[*f*]chromen-3-carbonitriles under solvent-free conditions. The catalyst exhibited excellent efficiency, providing the desired products in high yields (87–97%) within short reaction times. In addition, Fe_3_O_4_/MPL–[IL] can be easily separated from the reaction mixture using a magnet and can be reused five times without a significant loss in its catalytic activity. After the fifth cycle, the catalyst retained about 95% of its initial activity, confirming its high stability and reusability. Consequently, this catalyst system is highly suitable for benzo[*f*]chromen synthesis and shows great potential for broader catalytic applications due to its outstanding properties.

## Conflicts of interest

There are no conflicts to declare.

## Supplementary Material

RA-015-D5RA07067H-s001

## Data Availability

Data will be available on request. Supplementary information (SI) is available. See DOI: https://doi.org/10.1039/d5ra07067h.
