# Supplementary material for: Magnetic lignin-supported sulfonic acid ionic liquid: a novel catalyst for synthesis of 2-amino-4-aryl-4H-benzo[f]chromen-3-carbonitrile derivatives
Source: RSC Adv. 2025 Nov 25;15(54):46380–90. doi: 10.1039/d5ra07067h (PMC12646295; doi:10.1039/d5ra07067h)
Supplement: RA-015-D5RA07067H-s001 [file RA-015-D5RA07067H-s001.pdf]

Supplementary Information (SI) for RSC Advances.
This journal is © The Royal Society of Chemistry 2025

Supporting Information

Magnetic lignin-supported sulfonic acid ionic liquid: A novel catalyst for synthesis of 2-amino-4-aryl-4*H*-benzo[*f*]chromen-3-carbonitrile derivatives

Majidreza Gerami, Mahnaz Farahi*and Bahador Karami

2. Experimental

S1. Materials and Instrumentation

All the reagents, solvents, and chemicals were purchased from Merck, Sigma-Aldrich, and Fluka and were employed without additional purification. The infrared spectra (FT-IR) were recorded in the range of 400 to 4000 cm^{-1} using a Jasco 6300D spectrophotometer. Melting points were evaluated using an open capillary method with a Kruss Electrothermal KSB1N device. X-ray diffraction (XRD) characterization was performed on a Rigaku Ultima IV diffractometer. High-resolution images were captured utilizing field emission-scanning electron microscopy (FE-SEM) employing the TESCAN MIRA3 instrument. Elemental composition was assessed with an energy-dispersive X-ray Spectroscopy (EDX) module on a Tescan Vega system. Thermogravimetric analysis (TGA) was performed using a PerkinElmer STA 6000 apparatus. Magnetic properties were examined via Vibrating Sample Magnetometry (VSM) employing the Kavir Magnet system. Furthermore, transmission electron microscopy (TEM) images were examined using a Philips EM208S transmission electron microscope.

S2. Preparation of Fe_3O_4 Nanoparticles

$FeCl_3.6H_2O$ (1.15 g, 4.35 mmol) and $FeCl_2.4H_2O$ (0.43 g, 2.15 mmol) were dissolved in deionized water (35 mL). The solution was stirred under a nitrogen atmosphere at 85 °C for 40 minutes. After that, a 10 mL NaOH solution (25%) was slowly added until the mixture's color changed from brown to black. Stirring was maintained for another 30 minutes. The resulting material was separated using a magnet, rinsed thoroughly with deionized water, and then dried.[1]

S3. Synthesis of 3-Mercaptopropyl Lignin (MPL)

Lignin (0.5 g) was reacted with (3-mercaptopropyl) trimethoxysilane (3 mL) in dry toluene (30 mL). The resulting mixture was stirred at room temperature for 20 minutes and then refluxed for 24 hours. Next, the resulting suspension was filtered, washed with dry toluene, and then dried to yield the product known as 3-mercaptopropyl lignin (MPL).

S4. Preparation of Fe_3O_4/MPL Composite

In a round-bottom flask (100 mL), the synthesized nano-Fe_3O_4 (1 g) was well dispersed in an aqueous solution (30 mL) containing NaOH (7 wt%) and urea (12 wt%) by ultrasonication for 15 minutes. Subsequently, 3-mercaptopropyl lignin (MPL) (1 g) was added, and the reaction mixture was stirred at -12 °C for 2 hours. Afterward, the contents were kept frozen for one hour, allowing complete dissolution of 3-mercaptopropyl lignin. The resulting Fe_3O_4/MPL composite was then magnetically separated, rinsed with deionized water, and dried in a vacuum oven for 24 hours.

S5. Synthesis of Acidic Bronsted Ionic Liquid ([IL])

1,4-Butanesultone (6.1 g) was added to 1-vinylimidazole (4.7 g) at 0 °C, and the resulting mixture was stirred for 24 hours, during which it solidified. Next, the obtained solid was purified by rinsing with diethyl ether and subsequently dried under vacuum at 60 °C. In the next step, the synthesized salt was dissolved in water (5 mL), and an equal molar amount of sulfuric acid was

carefully added dropwise at 0 °C. The temperature of the solution was then slowly increased to 60 °C, and the reaction was stirred for 12 hours. At the end of the process, the final liquid product was washed using diethyl ether and dried under reduced pressure at 60 °C for 12 hours.

S6. Preparation of Fe_3O_4/MPL-[IL] Catalyst

Ultimately, a mixture of Fe_3O_4/MPL (0.5 g), the prepared ionic liquid (5 mmol), and azodiisobutyronitrile (AIBN) (5 mol%) in dry toluene (100 mL) was refluxed under a nitrogen atmosphere for 24 hours. Finally, the Fe_3O_4/MPL-[IL] catalyst obtained was washed with diethyl ether and then dried under vacuum conditions.

S7. Catalyst Recovery

For recycling, Fe_3O_4/MPL-[IL] (0.004 g) was magnetically separated from the reaction mixture, washed with water and ethanol, and dried before reuse. The catalyst retained its activity over multiple runs.
